# Supplementary material for: Comparing the physiques of elite Polish female and male swimmers training for short and long distances with their non-training peers – Is swimming a health-promoting sport?
Source: PLoS One. 2025 Dec 17;20(12):e0339041. doi: 10.1371/journal.pone.0339041 (PMC12711078; doi:10.1371/journal.pone.0339041)

PLOS ONE

Comparing the physiques of elite Polish female and male swimmers training for short and long distances with their non-training peers - is swimming a health-promoting sport?

--Manuscript Draft--

**Manuscript Number:**	PONE-D-25-30969
**Article Type:**	Research Article
**Full Title:**	Comparing the physiques of elite Polish female and male swimmers training for short and long distances with their non-training peers - is swimming a health-promoting sport?
**Short Title:**	Anthropometric characteristics of different distance swimmers and its health implications
**Corresponding Author:**	Anna Keska, Profesor<br>Józef Piłsudski University of Physical Education in Warsaw: Akademia Wychowania Fizycznego Jozefa Pilsudskego w Warszawie<br>Warszawa, POLAND
**Keywords:**	Anthropometry; swimming; gender; distance; Health
**Abstract:**	In athletes, anthropometric measures are widely used to prescribe desirable body weight, to optimize competitive performance, and to evaluate the effectiveness of various training regimens. However, it also seems interesting to find out which values of anthropometric indices showing a significant relationship with health risk characterise the top athletes, especially in popular sports. The aim of the study was to characterise the physique of female and male swimmers compared to their non-training peers and to determine to what extent it is influenced by training distance. Somatic measurements were taken in 30 female and 30 male top Polish swimmers. The subjects were divided into four groups, i.e. SDF (n=24) and SDM (n=24) groups comprising females and males training for short-distance, and LDF (n=6) and LDM (n=6) groups comprising those training for long-distance. The swimmers were compared with their non-training peers, 373 females and 155 males aged 20 to 30 years. BMI, WHR, BF%, BAI and Slenderness index were calculated to describe the athletes' physiques. Swimmers compared to non-swimmers had significantly lower BMI ($P < 0.001$), BF% ($P < 0.001$ for short-distance swimmers and $P < 0.01$ for long-distance swimmers, respectively) and WHR ($P < 0.001$). In contrast, swimmers had significantly higher Slenderness index values<br>($P < 0.001$). It was also found that the distance trained differentiates especially the physique of female swimmers, and that physique has a greater impact on athletic performance in short-distance swimmers. Monitoring the physique of top swimmers provides an insight into the type of adaptation to the training process, which in turn enables the identification of factors that determine sports success. Such observations also document the health benefits of swimming, allowing this form of physical activity to be promoted to the public.
**Order of Authors:**	Aleksandra Stachowicz
	Anna Keska, Profesor
	Katarzyna Milde
	Małgorzata Stachowicz
**Additional Information:**	
**Question**	**Response**
**Financial Disclosure**<br><br>Enter a financial disclosure statement that describes the sources of funding for the work included in this submission. Review	The author(s) received no specific funding for this work.

Powered by Editorial Manager® and ProduXion Manager® from Aries Systems Corporation

the submission guidelines for detailed requirements. View published research articles from *PLOS ONE* for specific examples.<br><br>This statement is required for submission and **will appear in the published article** if the submission is accepted. Please make sure it is accurate.<br><br>**Funded studies**<br>Enter a statement with the following details:<br>• Initials of the authors who received each award<br>• Grant numbers awarded to each author<br>• The full name of each funder<br>• URL of each funder website<br>• Did the sponsors or funders play any role in the study design, data collection and analysis, decision to publish, or preparation of the manuscript?<br><br>Did you receive funding for this work?	
**Competing Interests**<br><br>Use the instructions below to enter a competing interest statement for this submission. On behalf of all authors, disclose any competing interests that could be perceived to bias this work—acknowledging all financial support and any other relevant financial or non-financial competing interests.<br><br>This statement is required for submission and **will appear in the published article** if the submission is accepted. Please make sure it is accurate and that any funding sources listed in your Funding Information later in the submission form are also declared in your Financial Disclosure statement.<br><br>View published research articles from *PLOS ONE* for specific examples.	The authors have declared that no competing interests exist.

Powered by Editorial Manager® and ProduXion Manager® from Aries Systems Corporation

**NO authors have competing interests**<br><br>Enter: *The authors have declared that no competing interests exist.*<br><br>**Authors with competing interests**<br><br>Enter competing interest details beginning with this statement:<br><br>*I have read the journal's policy and the authors of this manuscript have the following competing interests: [insert competing interests here]*<br><br>* typeset	
**Ethics Statement**<br><br>Enter an ethics statement for this submission. This statement is required if the study involved:<br><br>• Human participants<br>• Human specimens or tissue<br>• Vertebrate animals or cephalopods<br>• Vertebrate embryos or tissues<br>• Field research<br><br>Write "N/A" if the submission does not require an ethics statement.<br><br>General guidance is provided below. Consult the submission guidelines for detailed instructions. **Make sure that all information entered here is included in the Methods section of the manuscript.**	Participation in the study was voluntary, which each person confirmed by signing a written consent. Ethical approval of the study was obtained from the Ethics Committee of the Józef Piłsudski University of Physical Education in Warsaw (No. SKE-01-15/2024).

**Format for specific study types**<br><br>**Human Subject Research (involving human participants and/or tissue)**<br>• Give the name of the institutional review board or ethics committee that approved the study<br>• Include the approval number and/or a statement indicating approval of this research<br>• Indicate the form of consent obtained (written/oral) or the reason that consent was not obtained (e.g. the data were analyzed anonymously)<br><br>**Animal Research (involving vertebrate animals, embryos or tissues)**<br>• Provide the name of the Institutional Animal Care and Use Committee (IACUC) or other relevant ethics board that reviewed the study protocol, and indicate whether they approved this research or granted a formal waiver of ethical approval<br>• Include an approval number if one was obtained<br>• If the study involved *non-human primates*, add *additional details* about animal welfare and steps taken to ameliorate suffering<br>• If anesthesia, euthanasia, or any kind of animal sacrifice is part of the study, include briefly which substances and/or methods were applied<br><br>**Field Research**<br><br>Include the following details if this study involves the collection of plant, animal, or other materials from a natural setting:<br>• Field permit number<br>• Name of the institution or relevant body that granted permission	
**Data Availability**<br><br>Authors are required to make all data underlying the findings described fully available, without restriction, and from the time of publication. PLOS allows rare exceptions to address legal and ethical concerns. See the PLOS Data Policy and FAQ for detailed information.	Yes - all data are fully available without restriction

A Data Availability Statement describing where the data can be found is required at submission. Your answers to this question constitute the Data Availability Statement and **will be published in the article**, if accepted.<br><br>**Important:** Stating 'data available on request from the author' is not sufficient. If your data are only available upon request, select 'No' for the first question and explain your exceptional situation in the text box.<br><br>Do the authors confirm that all data underlying the findings described in their manuscript are fully available without restriction?	
**Describe where the data may be found in full sentences. If you are copying our sample text, replace any instances of XXX with the appropriate details.**<br><br>• If the data are **held or will be held in a public repository**, include URLs, accession numbers or DOIs. If this information will only be available after acceptance, indicate this by ticking the box below. For example: *All XXX files are available from the XXX database (accession number(s) XXX, XXX.*).<br>• If the data are all contained **within the manuscript and/or Supporting Information files**, enter the following: *All relevant data are within the manuscript and its Supporting Information files.*<br>• If neither of these applies but you are able to provide **details of access elsewhere**, with or without limitations, please do so. For example:<br><br>*Data cannot be shared publicly because of [XXX]. Data are available from the XXX Institutional Data Access / Ethics Committee (contact via XXX) for researchers who meet the criteria for access to confidential data.*<br><br>*The data underlying the results presented in the study are available from (include the name of the third party*	All relevant data are within the manuscript.

and contact information or URL).<br>• This text is appropriate if the data are owned by a third party and authors do not have permission to share the data.<br><br>* typeset	
Additional data availability information:	

 Click here to access/download;Manuscript;Manuscript.docx

Full title: Comparing the physiques of elite Polish female and male swimmers training for short and long distances with their non-training peers - is swimming a health-promoting sport?

Short title: Anthropometric characteristics of different distance swimmers and its health implications

Aleksandra Stachowicz[1], Anna Kęska[2*], Katarzyna Milde[2], Małgorzata Stachowicz[3]

[1] Doctoral School, Józef Piłsudski University of Physical Education, Warsaw, Poland

[2] Department of Human Biology, Józef Piłsudski University of Physical Education, Warsaw, Poland

[3] Department of Water and Winter Sports, Józef Piłsudski University of Physical Education, Warsaw, Poland

* Corresponding author

E-mail: anna.keska@awf.edu.pl (AK)

Abstract

In athletes, anthropometric measures are widely used to prescribe desirable body weight, to optimize competitive performance, and to evaluate the effectiveness of various training regimens. However, it also seems interesting to find out which values of

anthropometric indices showing a significant relationship with health risk characterise the top athletes, especially in popular sports. The aim of the study was to characterise the physique of female and male swimmers compared to their non-training peers and to determine to what extent it is influenced by training distance. Somatic measurements were taken in 30 female and 30 male top Polish swimmers. The subjects were divided into four groups, i.e. SDF (n=24) and SDM (n=24) groups comprising females and males training for short-distance, and LDF (n=6) and LDM (n=6) groups comprising those training for long-distance. The swimmers were compared with their non-training peers, 373 females and 155 males aged 20 to 30 years. BMI, WHR, BF, BAI and Slenderness index were calculated to describe the athletes' physiques. Swimmers compared to non-swimmers had significantly lower BMI ($P < 0.001$), BF ($P < 0.001$ for short-distance swimmers and $P < 0.01$ for long-distance swimmers, respectively) and WHR ($P < 0.001$). In contrast, swimmers had significantly higher Slenderness index values ($P < 0.001$). It was also found that the distance trained differentiates especially the physique of female swimmers, and that physique has a greater impact on athletic performance in short-distance swimmers. Monitoring the physique of top swimmers provides an insight into the type of adaptation to the training process, which in turn enables the identification of factors that determine sports success. Such observations also document the health benefits of swimming, allowing this form of physical activity to be promoted to the public.

Introduction

Swimming is a low-impact, full-body workout that promotes cardiovascular health, improves lung capacity, strengthens musculoskeletal structures, and reduces the risk of joint injuries commonly seen in high-impact sports [1]. The health benefits of swimming are largely due to changes in body composition and overall physique [2]. Regular swimming training usually induces an increase in muscle mass, especially in the upper body muscles, which

generate most of the propulsive force and determine swimming speeds. Additionally, swimmers tend to develop a leaner physique with a low percentage of body fat, contributing to improved buoyancy and reduced drag in the water [3]. However, in the literature studies showing that swimmers have a higher body fat content than other athletes and non-athletes can also be found [4]. An important aspect of swimming training is its effect on body fat distribution. Unlike in many land-based sports, swimmers often retain a slightly higher level of subcutaneous fat, particularly around the torso, which may serve as insulation in the aquatic environment and aid in buoyancy [5]. As swimming is not only a popular sport, but also a type of physical activity that is important in the prevention of obesity and other chronic non-communicable diseases, it is of interest to determine how values of anthropometric indices showing a significant association with the risk of adverse health outcomes related to obesity characterise those who practice swimming compared to non-exercisers.

It is well known that the amount and distribution of body fat depends primarily on sex hormones. Males tend to have lower levels of body fat and a more central fat distribution compared to females [6]. Gender differences in fat content result in different its utilisation during exercise, as well as a different risk of developing diseases as a consequence of excess fat mass, particularly visceral fat [7]. Previous research has shown that gender influences changes in body composition in response to swimming training [8]. It therefore seems important to investigate to what extent swimming training modifies the amount and distribution of body fat in female and male swimmers.

Another factor that influences changes in physique in response to swimming training is the distance trained. Swimming can be performed at different distances. In pool swimming competitions the distances range from 50m to 1500m [9]. Research to date suggests that sprinters and long-distance swimmers often exhibit distinct morphological profiles that contribute to their specific competitive advantages. Sprinters focus on explosive power,

anaerobic capacity, and high-intensity resistance training to maximize speed and strength [10]. In contrast, long-distance swimmers emphasize endurance, aerobic efficiency, and sustained energy output, often incorporating high-volume training with a focus on pacing and technique conservation [11]. These differences in training regimens influence the development of specific somatic characteristics that improve hydrodynamics and overall performance in short- and long-distance swimming. Understanding this relationship can provide valuable information on optimising training, predicting performance and long-term health implications for elite swimmers.

This study aims to answer the question to what extent do the values of common anthropometric health-related indices in swimmers differ from their values in their non-training peers. An attempt was also made to examine the relationship between somatic characteristics and distance specialization among elite female and male swimmers.

Materials and methods

Participants

Recruitment for the study was conducted from 1 September to 30 October 2024. Participation in the study was voluntary, which each person confirmed by signing a written consent.

The study involved 30 female and 30 male swimmers aged 19-26 years (seniors), competing in pool swimming events. These were Polish athletes at the top of the national ranking lists at the time of the survey. The swimmers' performance has been expressed as FINA (Federation Internationale de Natation) point score (Ruiz-Navarro et al. [12]), calculated according the formula ‘1000 x (world record time/athlete time)^3 (s/s)^3’ from data available at https://www.swimrankings.net/?language=pl. The training length of the study participants averaged 12 years. The subjects were divided into four groups based on their gender and

distance specialisation. The SDF (n=24) and SDM (n=24) groups included female and male swimmers training for short distances, i.e. 50m and 100m, while the LDF (n=6) and LDM (n=6) groups included those training for long distances, i.e. 800m and 1500m. The general sport characteristics of the studied groups are shown in Table 1.

Table 1. Study groups of female (n=30) and male swimmers (n=30), Mean ± SD

	Training length, years	FINA points
SDF (n=24)	11.5 ± 2.3	768.5 ± 50.9
LDF (n=6)	11.8 ± 2.5	808.2 ± 50.9
SDM (n=24)	13.3 ± 2.0	833.5 ± 56.4
LDM (n=6)	12.5 ± 3.6	848.7 ± 45.3

Abbreviations: SDF- short-distance female swimmers, LDF- long-distance female swimmers, SDM- short-distance male swimmers, LDM- long-distance male swimmers.

The swimmers' physiques were compared with those of their non-training peers. The control group consisted of a total of 528 subjects (373 females and 155 males) aged 20 to 30 years (22.2 ± 3.0 years) studied by Kopiczko et al. [13].

Anthropometric measurements

Somatic measurements were taken by qualified anthropometrists according to the protocol of the International Society for the Advancement of Kinanthropometry [14]. Body height, body weight, waist and hip circumferences were measured. Height was measured to the nearest 0.1 cm and weight to the nearest 0.1 kg. Measurements were taken using a stadiometer and a medical scale. Waist and hip circumferences were measured to the nearest 0.1 cm using a non-stretch measuring tape. Waist circumference was measured at the mid-point between the lowest rib and the top of the iliac crest. Hip circumference was measured at the level of the greatest protrusion of the gluteal muscles. All measurements were taken in the morning.

Anthropometric indexes

Based on the above measurements, five indexes were calculated to describe the athletes' physiques. The first was the body mass index (BMI) calculated according to the formula ‘BMI = body mass /height^2 (kg/m^2)’ and interpreted according to the World Health Organization classification. According to it, a BMI < 18.5 indicates underweight, BMI values 18.5 -24.9 indicate normal weight, BMI values 25.0 - 29.9 indicates overweight and a BMI > 30.0 indicates obesity [15]. The waist-to-hip (WHR) index was calculated according to the equation ‘waist circumference/hip circumference (cm/cm)’. Its values were also interpreted using World Health Organisation guidelines, according to which in men android (visceral-abdominal) fatness was assumed for WHR values ≥ 1.0, and gynoid (gluteal-femoral) for WHR values < 1.0. In women, android fatness is found when WHR ≥ 0.85, and gynoid fatness when WHR < 0.85 [16].

Two indexes assessing body fat percentage were also used, i.e. the Body Fat Index (BF) and the Body Adiposity Index (BAI). BF was calculated according to the formula ‘BF = 1.2 × BMI + 0.23 × calendar age - 10.8 × gender - 5.4 (where men = 1; women = 0)’?? [17], and BAI as ‘BAI = hip circumference /body height$^{1.5}$ – 18 (cm/m$^{1.5}$)’ [18]. Interpretation of body fat content in athletes and non-athletes was made using the standards proposed by Gallagher et al. [19]. According to them in men, body fat content below 8% is considered low, values between 8% and 21% are considered normal, values between 21% and 26% are classified as overweight and values above 26% as obese. For women, a body fat percentage of 21% - 33% is considered normal, values below 21% are classified as underweight, values between 33% and 39% indicate overweight, while values above 39% are classified as obesity.

The formula ‘body height/ $\sqrt[3]{}$body weight (cm/kg$^{1/3}$)’ was used to calculate the Slenderness index [20]. The classification of body types on the basis of this index is as follows: in men, index values ≤ 41.54 indicate a bulky build, values 41.55 - 44.87 indicate a medium

build and values ≥ 44.88 indicate a slender build. In women, the cut-off values for the Slenderness index are: ≤ 41.47 blunt body build, 41.48 - 44.96 medium body build and ≥ 44.97 slender body build.

Statistical analysis

The normality of the distribution of the variables was tested using the Shapiro-Wilk test. The significance of differences between the groups separated on the basis of gender and distance trained was assessed using a two-factor analysis of variance ANOVA. The existence of a relationship between somatic parameters/indicators and swimmers' sporting level was ascertained from the Spearman correlation coefficient. A significance level of $P < 0.05$ was assumed for all tests. Statistical calculations were performed using STATISTICA v.13.

Results

We observed that swimmers compared to non-swimmers had a significantly higher body height (Fig 1). This difference applied to both short-distance swimmers ($P < 0.001$) and long-distance swimmers ($P < 0.05$). For body mass, we have established that short-distance swimmers had significantly lower ($P < 0.001$) and long-distance swimmers significantly higher ($P < 0.05$) body mass than non-trainees. The athletes were also characterised by significantly lower waist circumference ($P < 0.001$ for short-distance swimmers and $P < 0.05$ for long-distance swimmers, respectively) and significantly higher hip circumference compared to the control group ($P < 0.001$ regardless of distance trained).

Fig 1. Mean (± SE) values of anthropometric parameters and indexes of swimmers normalized to non-training groups.

*P < 0.05, **P < 0.01, ***P < 0.001 significant differences between the mean of the normalised values in short-distance and long-distance swimmers

^P < 0.05, ^^P < 0.01, ^^^P < 0.001 significant differences between the mean of the normalised values in the short-distance swimmers and the control group

#P < 0.05, ##P < 0.01, ###P < 0.001 significant differences between the mean of the normalised values in the long-distance swimmers and the control group

red line - mean values of the variables studied for non-trained females and males

We further found that swimmers compared to non-swimmers had significantly lower BMI (P < 0.001), BF (P < 0.001 for short-distance swimmers and P < 0.01 for long-distance ones, respectively) and WHR (P < 0.001). In contrast, swimmers had significantly higher Slenderness index values (P < 0.001). There were no significant differences between the compared groups (athletes/non-athletes) in the BAI (Fig 1).

Table 2 shows the results of the swimmers' anthropometric measurements. There were no significant differences in body height between the athlete groups. The type of distance specialisation, however, significantly differentiated body weight, but only in female athletes. Namely, long-distance swimmers had a significantly higher body weight compared to short-distance ones (P < .01). The length of the distance swum was also a factor differentiating waist and hip circumferences, but again only in the female swimmers. Significantly lower values of this parameters were recorded in the SDF group (P < 0.001).

Table 2. Somatic characteristics of the athletes, Mean ± SD

Measurement	SDF (n=24)	LDF (n=6)	SDM (n=24)	LDM (n=6)
Body height, cm	173.6 ± 5.3	174.0 ± 4.7	185.7 ± 5.8	184.9 ± 4.4
Body mass, kg	58.3 ± 5.8[a]	67.0 ± 4.2	78.5 ± 5.7	80.8 ± 3.8
Waist circumference, cm	71.1 ± 3.9[b]	78.3 ± 5.1	82.4 ± 5.8	83.2 ± 3.8
Hip circumference, cm	94.7 ± 4.5[b]	102.6 ± 3.1	97.7 ± 5.1	95.7 ± 4.0

Abbreviations: SDF- short-distance female swimmers, LDF- long-distance female swimmers, SDM- short-distance male swimmers, LDM- long-distance male swimmers.

[a] – significant differences between short-distance and long-distance female swimmers, P < 0.01.

[b] – significant differences between short-distance and long-distance female swimmers, P < 0.001.

The results of the two-factor ANOVA (gender x distance) are presented in Table 3. For BMI, there was a significant and large effect of the factor ‘gender’ ($F_{1,56}$ = 42.80; P < 0.001; η^2

= 0.43), as well as a significant and large effect of the factor 'distance' ($F_{1,56}$ = 22.23; P < 0.001; η^2 = 0.27). We also showed that higher BMI values characterised males (P < 0.001) and those specialising in long-distance swimming (P < 0.001). Moreover, a significant, large interaction effect of the factors 'gender x distance' ($F_{1,56}$ = 13.84; P < 0.001; η^2 = 0.20) was noted in the differential BMI. Using Tuckey's post hoc test, we demonstrated that short-distance female swimmers had a significantly lower BMI compared to the other female and male swimmers (P < 0.001).

Table 3. Comparison of anthropometric indexes of female and male swimmers training for short and long distances, Mean ±SD

	SDF (n=24)	LDF (n=6)	SDM (n=24)	LDM (n=6)	'gender'	'distance'	'gender' x 'distance'
BMI	19.3 ± 1.4	22.1 ± 0.6	22.8 ± 0.8	23.1 ± 0.8	***	***	***
BF	22.7 ± 1.6	26.2 ± 0.9	16.5 ± 1.0	16.8 ± 0.9	***	***	***
BAI	23.4 ± 2.0	26.7 ± 1.5	20.6 ± 2.2	20.1 ± 1.8	***	*	**
Slenderness index	44.8 ± 1.1	42.9 ± 0.5	43.4 ± 0.6	43.1 ± 0.6	*	***	**
WHR	0.7 ± 0.0	0.8 ± 0.0	0.8 ± 0.1	0.9 ± 0.0	***	NS	NS

Abbreviations: SDF- short-distance female swimmers, LDF- long-distance female swimmers, SDM- short-distance male swimmers, LDM- long-distance male swimmers.

*P < 0.05, **P < 0.01, ***P < 0.001 – statistical significance of the effects; NS - statistically non-significant difference.

A significant and large effect of the factor 'gender' ($F_{1,56}$ = 350.15; P < 0.001; η^2 = 0.86) and 'distance' ($F_{1,56}$ = 21.06; P < 0.001; η^2 = 0.27) was also found for BF (Table 3). It was observed that females (P < 0.001) and those specialising in long-distance swimming had higher BF values (P < 0.001). For BF, as with BMI, a significant, large interaction effect of 'gender x distance' factors was observed ($F_{1,56}$ = 14.92; P < 0.001; η^2 = 0.21). Post hoc analysis showed that the difference in BF values between female swimmers training for short and long distances was greater than between male swimmers training for different distances (P < 0.001).

The values of BAI were more strongly determined by gender ($F_{1,56} = 51.72$; $P < 0.001$; $\eta^2 = 0.48$) than by trained swimming distance ($F_{1,56} = 4.25$; $P = 0.044$; $\eta^2 = 0.07$). Higher BAI values were observed in females ($P < 0.001$) and in long-distance trainers ($P < 0.05$). In addition, there was a significant mean interaction effect of the factors 'gender x distance' ($F_{1,56} = 8.60$; $P = 0.005$; $\eta^2 = 0.13$). This allows to conclude that female short-distance as well as long-distance swimmers had significantly higher BAI values compared to male swimmers ($P < 0.001$), and that the distance swum affected the values of this index to a greater extent in female swimmers than in male ones (Table 3).

The effects of gender and distance on Slenderness index values in swimmers were opposite to those of BAI. Namely, there was a significant medium effect of the factor 'gender' ($F_{1,56} = 5.06$; $P = 0.028$; $\eta^2 = 0.08$) and a significant large effect of the factor 'distance' ($F_{1,56} = 17.03$; $P = 0.0001$; $\eta^2 = 0.23$) on the values of this indicator. We found that males were characterised by lower Slenderness index values compared to females ($P < 0.05$). In addition, lower Slenderness index values were characterised by those who trained in long-distance swimming ($P < 0.001$). A significant large interaction effect of 'gender x distance' factors was observed ($F_{1,56} = 10.89$; $P = 0.002$; $\eta^2 = 0.16$). On the basis of post hoc analysis, it was shown that female short-distance swimmers had significantly higher Slenderness index values compared to the other groups ($P < 0.001$).

Our results demonstrated that WHR values depended primarily on the gender of the athletes ($F_{1,56} = 38.08$; $P < 0.001$; $\eta^2 = 0.40$). Males had significantly higher WHR values ($P < 0.001$). In contrast, no significant effect was found for the factor 'distance' ($F_{1,56} = 1.31$; $P = 0.26$; $\eta^2 = 0.02$) and for the interaction 'gender x distance' ($F_{1,56} = 0.14$; $P = 0.74$; $\eta^2 = 0.003$) (Table 3).

It was noted that the predominant type of fat distribution in the female and male swimmers studied was the gynoid type. Only 10% of the female athletes and 3% of the male

athletes were characterised by android body fat distribution. Gender and distance specialisation did not significantly affect the prevalence of android and gynoid fatness among the athletes studied (G = 1.52; P = 0.68; R2 = 0.16).

Based on the correlation analysis, we noted a significant positive correlation between FINA points and BMI and WHR values. In contrast, female and male athletes characterised by higher FINA scores obtained lower BF, BAI and Slenderness index values. After taking into account the distance trained, these relationships remained statistically significant primarily in the group of short-distance swimmers (Table 4).

Table 4. Spearman correlation coefficient for somatic indexes and FINA points in male and female swimmers specialising in short and long distances.

	BMI	BF	BAI	Slenderness index	WHR
All swimmers (n=60)					
FINA	0.457*	-0.308*	-0.300*	-0.325*	0.328*
Short-distance swimmers (n=48)					
FINA	0.429*	-0.438*	-0.319*	-0.241	0.353*
Long-distance swimmers (n=12)					
FINA	0.393	-0.312*	-0.313	-0.068	0.158

*P < 0.05

Discussion

The main aim of this cross-sectional research was to characterise the physique of female and male swimmers compared to their non-training peers and to determine to what extent it is influenced by training distance. The study was conducted among top Polish athletes, whose somatic constitution was analysed on the basis of selected anthropometric indexes and comparison with non-training young adults. The general findings of our study are as follows: swimming maintains normal body fat content and health-promoting body fat distribution; the

distance trained differentiates especially the physique of female swimmers; and physique has a greater impact on athletic performance in short-distance pool swimmers.

Based on BMI values, we found that 89% of the swimmers were of normal weight (BMI between 18.5 and 24.9). However, in the group of short-distance female swimmers 29% were underweight (BMI < 18.5). The BMI values of the athletes, regardless of the distance trained, were significantly lower than the BMI of the non-athletes. This effect was certainly influenced primarily by body height, which was significantly higher in the short- and long-distance swimmers of both sexes than in the control group. In the case of the short-distance female swimmers, the significantly lower body mass also contributed to this. The relationship between body height and better swimming performance has been confirmed by many other researchers. Dopsaj et al. [21] also studied relations between swimming performance and the characteristics of body composition in highly trained swimmers. The research included 82 swimmers of international level (N = 46 male and N = 36 female athletes) from 8 European countries. The mean values of the basic anthropometric parameters, i.e. height, body mass and BMI recorded in this group of athletes were similar to our study and were 183.8 cm, 78.4 kg and 23.21 kgm2 for male swimmers and 171.5 cm, 63.1 kg and 21.45 kgm2 for female swimmers, respectively [21]. The aforementioned researchers further highlighted that female swimmers are taller, have lower body weight and BMI comparing with the results published previously [22]. Pla et al. [23] analysing height, weight, BMI and speed results for the top 100 international men and women over distances from 50 to 1,500m freestyle in the 2000-2014 seasons found that height was always positively correlated with speed with 95% probability. In this study, the authors proved that body height has a huge impact on swimming speed at all distances and for both genders. As an explanation, it is given that this is related to a greater arm span and a reduction of the drag coefficient [24]. In the case of body mass, our results differ from those available in the literature, according to which short-distance swimmers are characterised by higher body

mass, mainly due to more developed muscle mass than long-distance swimmers. However, in our participants' greater body mass of long-distance swimmers was associated with higher body fat, as will be discussed below.

Swimmers, compared to non-swimmers, were also characterised by significantly lower WHR values, which was a consequence of a significantly smaller waist circumference and a significantly larger hip circumference. The WHR values of both female and male swimmers indicated a gynoid type of fatness of their bodies. In explaining the benefits of this type of fat distribution in swimmers, it seems right to refer to the specific environment in which the exercise is performed. During swimming, the water around the swimmer is set in motion, which can be thought of as additional water mass to be overcome. People with a lower WHR have a lower added mass, which significantly reduces the energy cost of exercise. Similarly explained are the differences in the energy cost of swimming between males and females, who also differ in WHR values [25]. The gynoid type of fatness that characterises swimmers should also be considered beneficial from a health point of view. This type of fatness is characterised by less visceral fat compared to subcutaneous fat, which significantly reduces the risk of non-communicable diseases such as hypertension, diabetes, coronary heart disease and dyslipidaemia [26].

We observed that swimmers, irrespective of the distance trained, had significantly lower levels of body fat as expressed by the BF index compared to their untrained peers. Relating the swimmers' BF values to the healthy body fat norms proposed by Gallagher et al. [19], it should be noted that the values were within the ranges for lean individuals, for both women (21 - 33%) and men (8 - 21%). However, when comparing the fat percentage of the athletes participating in own study with the norms for swimmers proposed by Santos et al. [22] we found that the short-distance female swimmers had a BF at the 50th percentile (% body fat for this percentile in females is 23.29), while the long-distance ones were above the 75th percentile (% body fat

for this percentile in females is 25.15). Among male swimmers, regardless of the distance trained, the average body fat content exceeded the 75th percentile value (% body fat for this percentile in males is 15.02) for representatives of this sport determined by Santos et al. [22]. BAI values, the second fatness index used, did not differ between swimmers and their non-training peers. BAI indicated a higher body fat content than that indicated by BF, which is related to the fact that the calculation of BAI takes into account hip circumference, whose values in the swimmers, regardless of the distance trained, were higher than in the control group.

We also found that swimmers had significantly higher Slenderness index values compared to those who did not train. Relating the values of this index to norms, we concluded that among the short-distance female swimmers, 54% had a medium build (index values between 41.48 - 44.96) and 46% had a slender build (index values > 44.97), while all the long-distance female swimmers were characterised by a medium build. The Slenderness index values of the male swimmers, regardless of the distance trained, indicated a medium build.

This study also aimed to determine whether distance specialisation significantly differentiates the body physique of top male and female swimmers. Our results showed that such a relationship occurs mainly in female swimmers. Similar results were obtained by other researchers analysing the relationship between performance, body composition and somatotype in competitive swimmers [21]. Based on these findings, the authors stated that body composition and somatotype characteristics could serve as predictors of performance only for female athletes.

In fact, it turned out that short-distance female swimmers were characterised by significantly lower body weight, waist and hip circumferences, as well as lower body fat content compared to long-distance ones. So they had a slimmer physique. This is in agreement with reports by other authors on the relationship between body build and swimming speed.

Namely, there are significant evidence that fat reduction contributes to the development of speed and agility [27]. Lower body fat content probably results in lower body shape resistance (frontal area) and skin friction resistance. On the other hand, because body fat has a lower density than water, a higher body fatness may be beneficial due to increased buoyancy in water. This could explain the significantly higher body fat content in long-distance female swimmers than in short-distance ones.

Another reason for the higher body fat content in long-distance swimmers is the higher energy cost of prolonged exercise. The most energy-efficient substrate is fat, which, stored in subcutaneous adipose tissue, can additionally act as an insulating layer. A number of studies have shown that swimmers with more subcutaneous fat were able to withstand longer periods of cold water swimming compared to those with less body fat [28]. Among others, Scott et al. [29] observed that the range of mean subcutaneous adipose tissue thickness of competitive swimmers was 6-10 mm compared to an average value of 4 mm reported in control subjects. However, the relationship between body fat and improved swimming performance appears more complex. Roelofs et al. [30] observed that changes in % body fat during the competitive season of sprinters, distance swimmers and divers were not associated with changes in their performance. They showed that better performance of distance swimmers was significantly correlated with lean mass (those who had a greater increase in lean mass improved their performance more) [30].

Correlation analysis between the body composition indexes and FINA scores confirmed that the relationship between body composition and athletic performance in swimmers depends on the distance trained. The statistically significant correlations recorded in the whole group after its division by trained distance were essentially maintained in the group of short-distance swimmers. This seems to mean that in long-distance swimming, success is determined more by factors other than the athlete's physique.

Our study has several limitations. These include the use of calculated body fat to assess swimmers' physiques. However, this is how the amount of body fat was assessed in the population that constituted the control group in the present study. In future studies on the relationship between distance trained and swimmers' physique, it would furthermore be worth considering style specialisation.

Summarising the results of the study, it must be said that explaining the relationship between swimmers' body structure and the distance trained is complicated. This is because it requires taking into account not only the duration and intensity of the training, but also many factors related to the environment, including thermal conditions, drag coefficient, underwater currents, passive resistance, etc. factors that determine the energy cost of the effort. However, conducting such studies, especially if the participants are top athletes, provides important information about the impact of training on their physique, and thus on their sporting performance. This kind of analysis also document the health benefits of swimming, allowing this form of physical activity to be promoted to the public.

Acknowledgments

The research was carried out as part of the project named Monothematic Research Task No3, funded by the Józef Piłsudski University of Physical Education in Warsaw. Ethical approval of the study was obtained from the Ethics Committee of the Józef Piłsudski University of Physical Education in Warsaw (No. SKE-01-15/2024).

Author Contributions

Conceptualization: AS, AK, MS.

Formal Analysis: KM.

Funding Acquisition: AS, AK.

Investigation: AS, MS.

Methodology: AS, MS.

Project Administration: AS, AK, MS.

Resources: AS, MS.

Supervision: AS, AK.

Visualization: AS, AK, KM, MS.

Writing – Original Draft Preparation: AS, AK.

Writing – Review & Editing: AS, AK.

References

1. Hsu C, Krabak B, Cunningham B, Borg-Stein J. Swimming anatomy and lower back injuries in competitive swimmers: a narrative review. Sports Health. 2024;16(6):971-981. https://doi.org/10.1177/19417381231225213
2. Oja P, Memon AR, Titze S, Jurakic D, Chen ST, Shrestha N, et al. Health benefits of different sports: a systematic review and meta-analysis of longitudinal and intervention studies including 2.6 million adult participants. Sports Med Open. 2024;10(1): 46. . https://doi.org/10.1186/s40798-024-00692-x
3. Alves M, Carvalho DD, Fernandes RJ, Vilas-Boas JP. How anthropometrics of young and adolescent swimmers influence stroking parameters and performance? A systematic review. Int J Environ Res Public Health. 2022;19(5):2543. https://doi.org/10.3390/ijerph19052543
4. Dave P, Subhedar R, Mishra P, Sharma D. Body composition parameter changes among young male and female competitive swimmers and nonswimmers. Int J Med Sci Public Health. 2016;5(1):85-92. https://doi.org/10.5455/ijmsph.2016.2905201520

5. Born DP, Romann M, Lorentzen J, Zumbach D, Feldmann A, Ruiz-Navarro JJ. Sprinting to the top: comparing quality of distance variety and specialization between swimmers and runners. Front Sports Act Living. 2024;6:1431594. https://doi.org/10.3389/fspor.2024.1431594
6. Frank AP, de Souza Santos R, Palmer BF, Clegg DJ. Determinants of body fat distribution in humans may provide insight about obesity-related health risks. J Lipid Res. 2019;60(10): 1710-1719. https://doi.org/10.1194/jlr.R086975
7. Sam S. Differential effect of subcutaneous abdominal and visceral adipose tissue on cardiometabolic risk. Horm Mol Biol Clin Investig. 2018;33(1): 20180014. https://doi.org/10.1515/hmbci-2018-0014
8. Beckford SE, Webb MC. Anthropometric parameters: obesity and metabolic risks for non-communicable diseases among adolescent swimmers. West Indian Med J. 2018;67:31–38. https://doi.org/10.7727/wimj.2015.259
9. Rüst CA, Rosemann T, Knechtle B. Sex difference in age and performance in elite Swiss freestyle swimmers competing from 50m to 1,500m. Springer Plus. 2014;3:228. https://doi.org/10.1186/2193-1801-3-228
10. Yang L, Gao B, Chen Y, Xu Q, Zhou J, Tang Q. Comparing the effects of maximal strength training, plyometric training, and muscular endurance training on swimming-specific performance measures: a randomized parallel controlled study in young swimmers. J Sports Sci & Med. 2025;24(1):128-141. https://doi.org/10.52082/jssm.2025.128
11. Zamparo P, Cortesi M, Gatta G. The energy cost of swimming and its determinants. Eur J Appl Physiol. 2020;120(1):41-66. https://doi.org/10.1007/s00421-019-04270-y
12. Ruiz-Navarro JJ, López-Belmonte Ó, Gay A, Cuenca-Fernandez F, Arellano R. A new model of performance classification to standardize the research results in swimming. Eur J Sport Sci. 2022;23(4):478-488. https://doi.org/10.1080/17461391.2022.2046174

13. Kopiczko A, Cieplińska J, Stecka A. Ocena występowania zaburzeń stanu odżywienia, ogólnego otłuszczenia oraz dystrybucji tkanki tłuszczowej u kobiet i mężczyzn w wieku 20-30 lat. Med Og Nauki Zdr. 2015;21(4):339–345. (article in Polish) https://doi.org/10.5604/20834543.1186902
14. Marfell-Jones M, Olds T, Stew A, Carter L. International standards for anthropometric assessment. Australia: The International Society for the Advancement of Kinanthropometry; 2006.
15. World Health Organization. Global Strategy on Diet, Physical Activity, and Health. Geneva: WHO; 2004.
16. Waist circumference and waist-hip ratio: report of a WHO expert consultation. Geneva, 8-11 December 2008.
17. Deurenberg P, Weststrate JA, Seidell JC. Body mass index as a measure of body fatness: age- and sex-specific prediction formulas. Br J Nutr. 1991;65(2):105-114. https://doi.org/10.1079/bjn19910073
18. Bergman RN, Stefanovski D, Buchanan TA, Sumner AE, Reynolds JC, Sebring NG, et al. A better index of body adiposity. Obesity (Silver Spring). 2022;19(5):1083-1089. https://doi.org/10.1038/oby.2011.38
19. Gallagher D, Heymsfield SB, Heo M, Jebb SA, Murgatroyd PR, Sakamoto Y. Healthy percentage body fat ranges: an approach for developing guidelines based on body mass index. Am J Clin Nutr. 2000;72(3): 694-701. https://doi.org/10.1093/ajcn/72.3.694
20. Ozimek M, Nowak M, Krawczyk M, Zaborova V, Hubert J, Čepička L, et al. Somatic determinants of sports performance in Polish sprinters. JKES. 2021;96(31):35-47. https://doi.org/10.5604/01.3001.0015.7394

21. Dopsaj M, Zuoziene IJ, Milić R, Cherepov E, Erlikh V, Masiulis N, et al. Body composition in international sprint swimmers: Are there any relations with performance? Int J Environ Res Public Health. 2020;17(24): 9464. https://doi.org/10.3390/ijerph17249464
22. Santos DA, Dawson JA, Matias CN, Rocha PM, Minderico CS, Allison DB, et al. Reference values for body composition and anthropometric measurements in athletes. PLoS One. 2014;9(5):97846. https://doi.org/10.1371/journal.pone.0097846
23. Pla R, Leroy A, Massal R, Bellami M, Kaillani F, Hellard P, et al. Bayesian approach to quantify morphological impact on performance in international elite freestyle swimming. BMJ Open Sport Exerc Med. 2019;5(1): 1-7. https://doi.org/10.1136/bmjsem-2019-000543
24. Sammoud S, Nevill AM, Negra Y, Bouguezzi R, Chaabene H, Hachana Y. Allometric associations between body size, shape, and 100-m butterfly speed performance. J Sports Med Phys Fitness. 2018;58(5):630-637. https://doi.org/10.23736/S0022-4707.17.07480-1
25. Knechtle B, Dalamitros AA, Barbosa TM, Sousa CV, Rosemann T, Nikolaidis PT. Sex differences in swimming disciplines-can women outperform men in swimming? Int J Environ Res Public Health. 2020;17(10):3651. https://doi.org/10.3390/ijerph17103651
26. Abdi Dezfouli R, Mohammadian Khonsari N, Hosseinpour A, Asadi S, Ejtahed HS, Qorbani M. Waist to height ratio as a simple tool for predicting mortality: a systematic review and meta-analysis. Int J Obes (Lond). 2023;47(12):1286-1301. . https://doi.org/10.1038/s41366-023-01388-0
27. Cortesi M, Gatta G, Michielon G, Di Michele R, Bartolomei S, Scurati R. Passive drag in young swimmers: effects of body composition, morphology and gliding position. Int J Environ Res Public Health. 2020;17(6):2002. https://doi.org/10.3390/ijerph17062002
28. Price T, Cimadoro G, S Legg H. Physical performance determinants in competitive youth swimmers: a systematic review. BMC Sports Sci Med Rehabil. 2024;16:20. https://doi.org/10.1186/s13102-023-00767-4

29. Scott CG, Ducharme MB, Haman F, Kenny GP. Warming by immersion or exercise affects initial cooling rate during subsequent cold water immersion. Aviat Space Environ Med. 2004;75(11): 956-963. PMID: 15558995.

30. Roelofs E, Emith-Ryan A, Trexler E, Hirsch K. Seasonal effects on body composition, muscle characteristics, and performance of collegiate swimmers and divers. J Athl Train. 2017;52(1): 45–50. https://doi.org/10.4085/1062-6050-51.12.26

Click here to access/download;Figure;Fig 1..tif

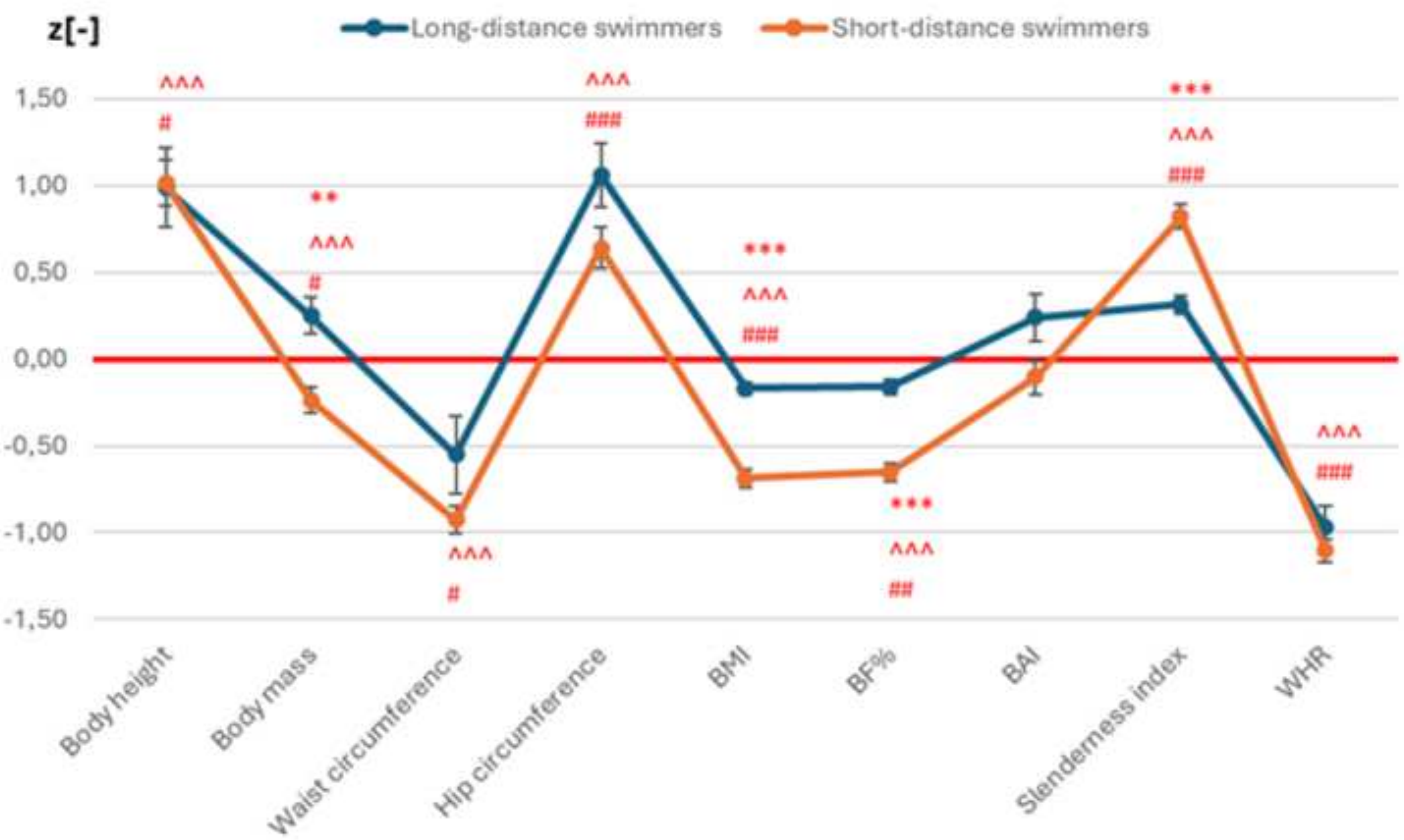

Supplement: Attachment — Submitted filename: PONE-D-25-30969_reviewer.pdf [file pone.0339041.s001.pdf]
