# Supplementary material for: Comparing the physiques of elite Polish female and male swimmers training for short and long distances with their non-training peers – Is swimming a health-promoting sport?
Source: PLoS One. 2025 Dec 17;20(12):e0339041. doi: 10.1371/journal.pone.0339041 (PMC12711078; doi:10.1371/journal.pone.0339041)
Supplement: Attachment — Submitted filename: Response to Reviewers.docx [file pone.0339041.s003.docx]

**Response to Reviewer 1**

Dear Reviewer,

We sincerely appreciate your comprehensive review and constructive feedback on our manuscript. Your comments have been extremely helpful in identifying areas where our manuscript needs improvement.

Below are the specific responses and explanations of the revisions for each of the comments raised.

1. Sample size imbalance: The long-distance swimmer subgroups (n=6 each) are underpowered, limiting the generalizability of some conclusions. The small sample size should be acknowledged more clearly in both the discussion and limitations section. Consider discussing whether any power analysis was conducted.

We fully agree with the reviewer's comment that the small number of long-distance swimmers is a limitation of this study. This comment has been added to the section ‘limitations of this study’ in the manuscript. At the same time, we would like to clarify that among top Polish swimmers, who were the only ones included in the study, there were significantly fewer people training for long distances. This distance is less popular not only in Poland. We would also like to emphasise that the small number of long-distance swimmers was due to compliance with the criteria for inclusion in the study. The participants were swimmers who competed in the national championships during the study and agreed to take part in the research.

2. Body fat estimation: The use of formula-based estimations (e.g., BF%, BAI) instead of direct measures like DEXA or BIA may reduce accuracy. While this limitation is mentioned, it should be more clearly emphasized, especially regarding how it might affect comparisons with reference norms.

Once again, we fully agree with the reviewer's opinion that calculating body fat content is a much less accurate method than measuring it using methods such as BIA or DEXA. With this in mind, we used the standards proposed by Gallagher et al. to interpret the results obtained. These authors established percentage body fat levels corresponding to BMI thresholds for underweight (<18.5), overweight (≥25) and obesity (≥30) based on gender-adjusted formulas that estimate relative body fat content based on BMI and other potential independent variables such as age and ethnicity. These formulas have been validated using reference methods for assessing total body fat content.

3. Swimming stroke not accounted for: Different strokes (freestyle, breaststroke, etc.) may influence body composition differently. Although this is briefly mentioned in the limitations, adding a sentence on how stroke style could confound findings would strengthen the manuscript.

Thank you for pointing this out. We have added this sentence to the limitations of the study section.

*Swimming style can affect body composition, as different styles require different amounts of energy and involve different training patterns. For example, freestyle and butterfly are typically less energy-intensive than breaststroke, which may result in higher body fat content in athletes who specialise in these styles. Therefore, further research is needed, in which, in addition to the distance covered, the training style will also be taken into account in the analysis of body composition.*

4. Clarity of subgroup comparisons: In several parts of the results and discussion, findings from male and female swimmers, or short- and long-distance groups, are compared. It would help the reader if the authors clarified whether these comparisons are statistically supported or descriptive.

Any differences between the compared subgroups, distinguished both in terms of the gender of the study participants and the trained distance, were statistically verified.

Once again, we thank you for your valuable time and feedback. We look forward to your further comments and hope that our revisions meet your expectations.

**Response to Reviewer 2**

We are truly grateful for your thorough review and valuable suggestions regarding our manuscript. Your feedback has been instrumental in highlighting aspects that required refinement.
Below, we provide detailed responses and explanations of the revisions made in accordance with each of your comments.

1. Please consider presenting the “p” in italics and presenting more numerical results in the abstract.

Thank you for this tip. We have made the appropriate changes to the manuscript.

2. Please consider developing the introduction section, supporting the text with references, and clearly highlighting the research gap before the presentation of the aims of the study. Please consider standardizing the paragraph size to improve readability (8-12 lines suggested throughout the manuscript).

As suggested by the reviewer, we have added information to the Introduction justifying the purpose of the research and we have revised the length of the paragraphs throughout the manuscript.

3. Please revise the citation format (not only in this line, throughout the manuscript).

Thank you for this tip. We have made the appropriate changes to the manuscript.

4. Please consider characterizing the sample. Some examples: Swimmer's training routines (in-water / dryland) and background. It is also relevant to describe the inclusion and exclusion criteria. Moreover, all data collection and statistical procedures should also be described in detail – e.g. sample power (GPower used?).

Following the reviewer's advice, we have supplemented the information on the participants' training and the criteria for inclusion and exclusion from the study.

*Over the study period, all athletes were completing 8-10 in-water training sessions a week (90–120 minutes), swimming a distance of 6,000–14,000 m per day. They also underwent land training 2 to 4 times a week (60–90 minutes), during which they performed strength, mobility and stabilisation exercises.*

*The inclusion criteria were high athletic level and participation in training sessions during the study, while exclusion criteria were injury preventing training or lack of consent to participate in the study.*

*The participants underwent identical assessment session in the morning hours. Before the measurements, they were well rested, after fasting overnight, and properly hydrated. They were asked to avoid intense physical activity for at least 12 hours before the measurements. All tests were conducted in a well-ventilated test room with controlled temperature and humidity by a trained person with many years of experience.*

5. Please consider reformulating the results section. It is too long, which difficult for readers' comprehension.

Thank you for bringing this to our attention. The description of the results has been shortened. We hope that in its current form it is more comprehensible.

6. Please consider shorter paragraphs in this section (Discussion) to improve readability, addressing the study rationale, and at the end of the section, presenting the study limitations and suggestions for future research.

The Discussion section has been modified in accordance with the reviewer's suggestions.

7. Please double-check the references format.

The formatting of the references has been corrected.

Once again, we sincerely thank you for your thoughtful and constructive feedback. We hope that the revisions made will meet the reviewer’s expectations and lead to the acceptance of our manuscript.

On behalf of all authors,

yours sincerely,

Anna Kęska
