# Supplementary material for: Comparing the physiques of elite Polish female and male swimmers training for short and long distances with their non-training peers – Is swimming a health-promoting sport?
Source: PLoS One. 2025 Dec 17;20(12):e0339041. doi: 10.1371/journal.pone.0339041 (PMC12711078; doi:10.1371/journal.pone.0339041)
Supplement: Attachment — Submitted filename: Response_to_Reviewers_auresp_2.docx [file pone.0339041.s004.docx]

**Response to Reviewer 2**

Dear Reviewer,

Thank you very much for your further comments on our manuscript. Below are the specific responses and explanations of the revisions for each of the comments raised.

1. L234 – Please place the units in the variables and describe the variables in full in the table footnote.

Thank you for this tip. We have made the appropriate changes to the manuscript.

2. L283-284 – Please revise the “p” format – Suggested in lowercase and italics throughout the manuscript. Please carefully check all format details throughout the manuscript, considering the journal template and instructions for authors.

As suggested by the reviewer, we have corrected format of ‘p’ throughout the manuscript.

3. L317 – Please consider placing the citation number close to the name of the authors. Please revise all manuscript considering this detail.

Thank you for this suggestion. We have made the appropriate changes to the manuscript.

4. L331-335 – Please consider standardizing the paragraphs size (8-12 lines suggested), aiming to improve readability. In these lines the paragraphs is too short, in other too long.

Following the reviewer's advice, we have attempted to standardise the length of the paragraphs while maintaining their substantive consistency.

5. L501 – Please revise ref 17 format. Please carefully revise all references format.

Thank you for this tip. We have rechecked and corrected all references.

Thank you very much for these comments. We hope that the revised manuscript will be accepted by the reviewer.

On behalf of all authors,

yours sincerely,

Anna Kęska
